# Four new Caribbean *Sigambra* species (Annelida, Pilargidae), and clarifications of three other *Sigambra* species

**DOI:** 10.3897/zookeys.893.39594

**Published:** 2019-12-02

**Authors:** Sergio I. Salazar-Vallejo, Alexandra E. Rizzo, J. Ángel de León-González, Kalina M. Brauko

**Affiliations:** 1 Depto. Sistemática y Ecología Acuática, El Colegio de la Frontera Sur, Chetumal, QR, México El Colegio de la Frontera Sur Chetumal Mexico; 2 Laboratório de Zoologia de Invertebrados, Universidade do Estado do Rio de Janeiro, Maracanã, Rio de Janeiro, Brazil Universidade do Estado do Rio de Janeiro Rio de Janeiro Brazil; 3 Laboratorio de Biosistemática, Facultad de Ciencias Biológicas, Universidad Autónoma de Nuevo León, Monterrey, NL, México Universidad Autónoma de Nuevo León Monterrey Mexico; 4 Benthic Laboratory, NEMAR, Federal University of Santa Catarina, Florianópolis, SC, Brazil Federal University of Santa Catarina Florianópolis Brazil

**Keywords:** dorsal hooks, Fritz Müller, key to species, morphology, polychaetes, taxonomy

## Abstract

*Sigambra grubii* Müller, 1858 has been reported from many different coastal environments in Brazil and the Grand Caribbean. However, more than one species was thought to be included under this species group name. After the study of several subtle and consistent differences in specimens fitting the description *S. grubii*, a new Grand Caribbean species is herein recognized and described as *S. hernandezi***sp. nov.** Further, the study of other *Sigambra* specimens prompted the examination of type specimens of *S. bassi* (Hartman, 1947), and of *S. wassi* Pettibone, 1966 to clarify some morphological features, and three other new species are recognized and newly described: *S. diazi***sp. nov.** and *S. ligneroi***sp. nov**. from the southeastern Caribbean (Venezuela), and *S. olivai***sp. nov.** from the northwestern Caribbean (México). Morphological features are also clarified for *S. grubii* by comparison with specimens from the type locality, Florianópolis, Brazil, and with type specimens of *S. bassi* from Florida (U.S.A.), and non-type specimens of *S. wassi* from Virginia (USA). A key to identify all species of *Sigambra* is also included.

## Introduction

Fritz Müller (1822–1897) was regarded by Darwin as the ‘Prince of Observers’ after his careful studies of many different plant and animal groups in Santa Catharina, Brazil (Hartfelder 2019). Müller’s research interests resulted in 263 published articles, which are certainly remarkable even after current standards. Müller wrote two papers dealing with polychaetes (Hartman 1951): one was part of his series of arguments and examples supporting Darwin, where he referred to an amphinomid living on goose barnacles; the other one was a report about the polychaetes found from Santa Catharina Island, Florianópolis, Brazil (Müller 1858). A panoramic description of the main benthic and pelagic organisms was part of a letter dated November 1856 and sent to his brother Hermann (Möller 1921: 9). Müller (1858) proposed eight new genera and described nine new species from Santa Catharina Island. Six of his genus-group names are listed as valid in WoRMS: *Cherusca*, *Glycinde*, *Hermundura*, *Isolda*, *Magelona*, and *Sigambra*. *Sigambra grubii*, the type species for the genus, was briefly described based on a living specimen in 13 text lines and three figures of the anterior end, parapodium, and dorsal hook.

Hartman (1947: 483) hesitated about recognizing *Sigambra*; she thought it could be the same as *Ancistrosyllis* McIntosh, 1879, and indicated that its status should be solved by the study of topotypes (non-type specimens from the type locality). She later regarded *Sigambra* as questionably the same as *Ancistrosyllis* (Hartman 1959: 195). Pettibone (1966: 156, 157, 179 ff) reinstated *Sigambra* and since then, it has been recorded in many localities along Brazilian coasts. Currently, *S. grubii* is regarded among the most abundant marine benthic polychaete species along south and southeastern Brazil, in sediments of up to 150 m depth (Rohr and Almeida 2006). However, it has been recorded in over 110 studies along Brazilian coasts (Amaral et al. 2013) from estuarine areas to marine deep-water sediments (1000–3000 m), including the northern Amazonian mangrove region (Cutrim et al. 2018, Ribeiro et al. 2018). Given such a wide bathymetric range, more than one species might be included under the same name. Further, because of the lack of a clarification of the morphology of *S. grubii*, some other unlikely species records have been reported in literature from the region. For example, de Almeida et al. (2012) recorded *S. pettiboneae* Hartmann-Schröder, 1979, a species originally described from Australia, from Santa Catharina State, Brazil.

The present study was prompted by the finding of *Sigambra* specimens in brackish waters of Tamiahua Lagoon in the Mexican coastal states of Veracruz and Quintana Roo, and the need to clarify the details of some widely distributed *Sigambra* species including the amphiamerican *S. bassi* (Hartman, 1947). Type material of *S. bassi*, *S. wassi* Pettibone, 1966, and topotype specimens of *S. grubii* were studied and compared to specimens from the Grand Caribbean and southern Brazil. We found some subtle and consistent differences in the Grand Caribbean specimens, and they are herein described as new. We also provide additional observations for the topotypes to clarify their differences. A key to identify all species in the genus is also included.

## Materials and methods

Tamiahua Lagoon is on the Mexican Gulf of México coast, in the northern part of Veracruz. After a heavy rain season in 1999 a mass benthic mortality or defaunation occurred, prompting a study on the recovery of polychaete benthic communities. Six systematic samplings were carried out at the site, through a network of four transects each with four stations, during November 1999, March, August, and November 2000, July 2001, and February of 2002 (Sánchez-Hernández 2009). Sediments were taken with an Eckman dredge and washed through a 0.5 mm mesh screen; polychaetes were fixed in a 10% formalin solution and preserved in 70% ethanol.

Material from the southern Brazilian coast was collected from a non-vegetated tidal flat adjacent to salt marshes and mangroves in Pontal da Daniela (27°27'11"S, 48°31'47"W), Santa Catharina Island, Florianópolis. Sediment samples were taken during low tide with a manual PVC corer (10 cm diameter x 15 cm depth), in summer and winter of 2018. Sediments were washed through a 0.5 mm mesh. Specimens were removed and then fixed in a 6% formalin solution in sea water, with Rose Bengal. Specimens were later sorted and transferred to 70% ethanol.

Specimens were often twisted and measuring their length or counting their chaetigers became problematic. For the length, the specimen was carefully set along a ruler and the width measured at approximately chaetiger 10 including dorsal cirri. For twisted specimens, the number of chaetigers is given as an estimate (ca.) when they exceeded approximately 90. Start of dorsal hooks was determined from specimens mounted in a 1:1 solution of glycerin and 70% ethanol and scanned with a compound microscope. For indicating the relative size of median to lateral antennae, specimens were observed in profile, because tips of the median antenna were often damaged. If possible, these appendages were measured directly with a mini scale such as BioQuip 4828M. The first presence of dorsal hooks depended on the visibility of the hook, or at least on their broken handles, when the specimens were viewed along their dorsal parapodial surfaces.

Specimens were often temporally stained with Methyl Green or Shirlastain-A; the latter was especially useful for detecting papillae in the basal pharyngeal ring. Digital photographs were made in both stereo- and compound microscope, and for some species, SEM micrographs were made in the El Colegio de la Frontera Sur (ECOSUR) facility. Photo series were compressed by using HeliconFocus software. The sequence of species described in Systematics is alphabetical.

Specimens are deposited in the following collections:

**ECOSUR**El Colegio de la Frontera Sur, Chetumal, México.

**LACM** Allan Hancock Polychaete Collection, Los Angeles County Museum of Natural History, Los Angeles, U.S.A.

**UANL**Polychaete Collection, Facultad de Ciencias Biológicas, Universidad Autónoma de Nuevo León, Monterrey, México.

**USNM** National Museum of Natural History, Smithsonian Institution, Washington, USA

## Systematics

### Order Phyllodocida Dales, 1962

#### Suborder Nereidiformia Glasby, 1993

**Family Pilargidae de Saint-Joseph, 1899**

**Subfamily Pilarginae de Saint-Joseph, 1899**

##### Sigambra

Taxon classificationAnimaliaAnnelidaPilargidae

Müller, 1858

1F42F53E-CA87-51D0-9013-EC5E426FA92A

Sigambra Müller, 1858: 214; Pettibone 1966: 179 (reinstated); Licher and Westheide 1997: 2 (key to species); Nishi et al. 2007: 65 (table with characters of all species).

###### Type species.

*Sigambra grubii* Müller, 1858, by monotypy.

###### Diagnosis.

Pilarginae with body depressed, usually obconic. Prostomium with three antennae, longer than palps; palps biarticulate. Tentacular cirri as long as half width of tentacular segment. Parapodia biramous. Dorsal and ventral cirri foliose to tapered, dorsal ones usually longer than ventral ones. Notopodia include dorsal hooks along many segments, sometimes with accessory capillaries. Neuropodia with shorter pectinates, medium-sized denticulates, and longer finely denticulate capillaries, often twisted distally.

###### Remarks.

*Sigambra* species were reviewed by Licher and Westheide (1997), and they modified the orthography for the type species, using *grubei* instead of *grubii*, as originally introduced, and included a key to species. However, *Sigambra grubii* does not need an orthographic modification. As was customary in the times, Müller (1858) did not include etymologies for his new taxa. Licher and Westheide (1997: 4) referred to article 31a of the code (ICZN 1985: 61, 1999: 37) in an aim to change the orthography for the specific epithet to *grubei*. This was incorrect because of three reasons: First, they apparently misunderstood the corresponding examples for the same section in the code, especially the last one (reiterated in the most recent edition): ‘Cuvier, if Latinized to Cuvierius, gives *cuvierii*.’ Second, the original epithet was not modified by either De Quatrefages (1866: 89), nor Pettibone (1966: 182), both had a good knowledge of Latin, and Licher and Westheide (1997: 3) referred to these publications. And third, Licher and Westheide overlooked the proposals of two other species using the same epithet (*Onuphis grubii* von Marenzeller, 1866, and *Arenicola grubii* Claparède, 1869), which would underline the fact that the original orthography was correctly formed once the last German name was Latinized. Consequently, the original orthography must be retained.

*Sigambra* Müller, 1858 resembles *Ancistrosyllis* McIntosh, 1879 by having dorsal hooks above the dorsal cirri (Salazar-Vallejo and Rizzo 2009: 431). They differ by the relative size of the antennae, tentacular and dorsal cirri, and body papillation. In *Sigambra* these appendages are long, foliose to tapered, usually antennae are longer than palps, and the integument is mostly smooth, whereas in *Ancistrosyllis* appendages are short, usually digitate, palps are longer than antennae, and integument is mostly papillate.

Diagnostic features for all the then known species were tabulated by Nishi et al. (2007). Specific diagnostic features are included below in the key to species. Anterior end features include the relative length of median antennae, the length of tentacular segment and presence of modifications along its anterior margin, the presence of ventral cirri on chaetiger 2, and of a constriction on anterior chaetigers. Parapodial features include the relative size of dorsal and ventral cirri, the start of dorsal hooks and their presence along body, and the type of neurochaetae. For the pharynx, the number of marginal papillae is especially useful. There are two patterns regarding the start of dorsal hooks. In the first, their start tends to be more or less stable, with a very small variation (2–4 chaetigers) disregarding variations in total size or number of chaetigers. In the other pattern, notohooks start at an earlier chaetiger in smaller specimens, and they are apparently displaced posteriorly during ontogeny, such that larger specimens will have dorsal hooks from a more posterior chaetiger. Further, notohooks along a few anterior chaetigers are often embedded in the notopodial bases, such that it is necessary to observe the specimen under a compound microscope to precise on which chaetiger notohooks arise. This implies that a series of specimens of different size, collected from the same date, and from similar depths, and sediment types, should be analyzed before deciding which alternative to follow in the key below. If available, size ranges were included in parenthesis to help guide decisions in the key, following Nishi et al. (2007).

There are four other potentially useful characters. First, the prostomial dorsal surface between the palps (interpalpal region) can be characterized by its anterior margin as blunt or depressed, and by the lateral depressions being widened posteriorly, or rectangular if the lateral depressions are more or less parallel. Second, in some species there is a deep antennal furrow for each lateral antenna; they can be easily noted if distinct, or as indistinct if they are difficult to see; further, antennal furrows are often narrower medially, and then they can diverge slightly, being almost parallel, or markedly divergent. Third, in some species, the ventral cirri can be short, not reaching neuropodial lobes tips, whereas in other species, ventral cirri can reach and even surpass neurochaetal lobes in medial or posterior parapodia. Fourth, the number of posterior chaetigers without hooks: in posterior chaetigers the dorsal hooks tend to be more exposed and are usually larger than those present in medial chaetigers or larger than parapodial lobes.

##### Sigambra bassi

Taxon classificationAnimaliaAnnelidaPilargidae

(Hartman, 1947)

C2CBDE97-B0ED-510E-8ABD-1D644D003B44

Fig. 1

Ancistrosyllis bassi Hartman, 1945: 6 (contents), 9 (substrate), 15 (non-diagnostic characters), nomen nudum.
Ancistrosyllis bassi Hartman, 1947: 501–504, pl. 61, figs 1–7; Hartman 1951: 36–38, pl. 11, figs 1–6 (figures rearranged from Hartman 1947).
Sigambra bassi : Pettibone 1966: 186, fig. 16, comb. nov.

###### Type material.

***Holotype*** (LACM 142), Gulf of México, southwestern Florida, Lemon Bay, Chadwick Beach (26°55'25"N, 82°21'40"W), sandy shore, low tide, 17 Jan. 1938, O. Hartman, coll. ***Paratype*** (LACM 1549), northwestern Atlantic, Beaufort, Bogue Sound, North Carolina, near US Fisheries Laboratory on Pivers Island (34°43'11"N, 76°40'19"W), sandy shore, 6 Jun. 1940, O. Hartman, coll.

###### Additional material.

**Northeastern Pacific, Los Angeles**. Non-type specimen (LACM 6614), opposite Berth 79, at old Municipal Fish Market, in mid-channel, Sta. LA-26 (33°33'17"N, 118°16'34"W), 12 m, black mud, 14–15 Jun. 1954, D.J. Reish et al. coll.

###### Clarification.

**Figure 1.**
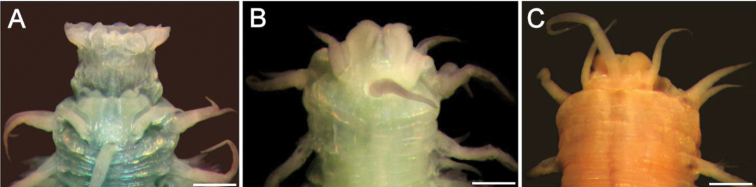
*Sigambra bassi* (Hartman, 1947) **A** holotype (LACM 142), anterior end, dorsal view **B** paratype (LACM 1549), anterior end, dorsal view **C** non-type specimen (LACM 6614), anterior end, dorsal view. Scale bars: 0.3 mm (**A, C**), 0.2 mm (**B**).

The holotype (Fig. 1A) was found in the Gulf of México side of Florida, whereas the paratype (Fig. 1B) was found in North Carolina. Both specimens are colorless, without any trace of pigmented glands. An additional specimen (Fig. 1C) was included in the same species, but it was collected in Los Angeles Harbor, and it has some brownish glands in both dorsal tentaculophores and dorsal cirri of chaetiger 1. There are some other differences between these specimens. For example, in the holotype the interpalpal area is slightly projected anteriorly and its margins are slightly divergent posteriorly, the lateral antennae are three times longer than wide, and the lateral antennal depressions are markedly divergent and expanded, such that the anterior region is wider than posterior one. In the paratype the interpalpal area is tapered, reduced anteriorly, with its margins expanded posteriorly, the lateral antennae are 4 times longer than wide, and the lateral antennal depressions diverge laterally such that the anterior region is shorter than the posterior one. The non-type specimen has a truncate interpalpal region, with its anterior margin slightly bilobed, and margins more or less parallel, the lateral antenna are more than 12 times longer than wide, and the lateral antennal depressions are barely distinct, visible only along the inner side of each antenna.

###### Remarks.

If these specimens are conspecific, the species would have a Gulf of México-Atlantic and Eastern Pacific distribution, which is untenable, for the following reasons. A strong genetic discontinuity between Gulf and Atlantic populations has been noted for specimens living in Florida (Soltis et al. 2006, Bijak et al. 2018), and amphi-American species have been progressively recognized as including more than one distinct species upon morphological features and genetic markers combined (Carrera-Parra and Salazar-Vallejo 2011), or even after the analysis of morphological differences (Conde-Vela and Salazar-Vallejo 2015). Consequently, *S. bassi* should be regarded as a northwestern Atlantic species restricted to the Gulf of México; new names and descriptions will be provided for the specimens from Beaufort, NC, and Los Angeles, California in a subsequent publication. Chances are, however, that the same species might extend from the Gulf of México to Beaufort, NC On the other hand, it should be noted that specimens from San Francisco have been regarded as belonging to a different species since 2013 (Norris 2013).

##### Sigambra diazi sp. nov.

Taxon classificationAnimaliaAnnelidaPilargidae

3069AF52-6C22-5A21-83CD-B37B96B0E7BC

http://zoobank.org/B8CC2C1F-6108-47CC-8427-08AF56D99593

Fig. 2

Sigambra tentaculata : Liñero-Arana and Díaz-Díaz 2005: 68–69, fig. 2 (**non**Treadwell 1941).

###### Type material.

***Holotype*** (ECOSUR 214) and ***paratype*** (ECOSUR 215), southern Caribbean, Venezuela, Laguna de Chacopata (10°39'50"N, 63°48'30"W), 1.5 m, sediments, 15 May 2000, O. Díaz-Díaz, coll.

###### Diagnosis.

*Sigambra* with median antenna reaching chaetiger 5–7, twice as long as laterals; tentacular segment four times wider than long, anterior margin smooth; dorsal cirri larger than ventral ones; chaetiger 2 without ventral cirri; notopodia with dorsal hooks and capillaries from chaetiger 4–5; median and posterior chaetigers with ventral cirri reaching neuropodial lobes tips; pharynx with 13–16 marginal papillae.

###### Description.

**Figure 2.**
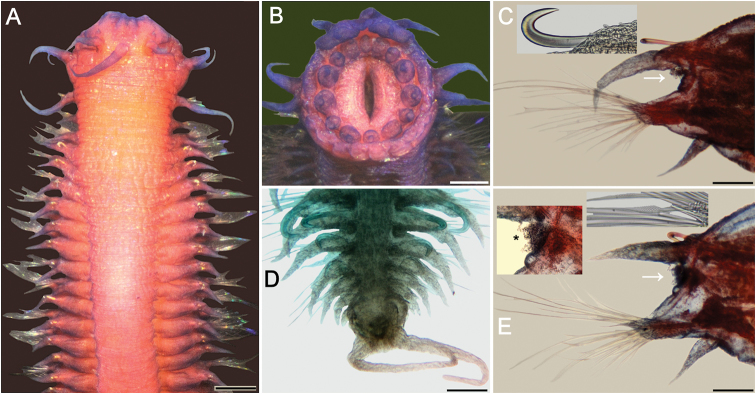
*Sigambra diazi* sp. nov. **A** holotype (ECOSUR 214), stained with Shirlastain-A, anterior region, dorsal view **B** same, frontal view of pharynx **C** Chaetiger 16, right parapodium, anterior view (arrow points to gonopore; inset: notohook) **D** Paratype (ECOSUR 215), stained with methyl green, posterior end, dorsal view **E** Chaetiger 40, right parapodium, anterior view (arrow points to gonopore; inset: hypertrophied gonopore with sperm, indicated with an asterisk, and central pectinate neurochaetae). Scale bars: 0.3 mm (**A, B**), 150 µm (**C**), 0.1 mm (**D**), 90 µm (**E**).

Holotype (ECOSUR 214) an anterior fragment, 7.5 mm long, 1.5 mm wide, 41 chaetigers, right parapodia of chaetigers 16 and 40 removed for observing parapodial features. Body obconic, cylindrical along chaetigers 1–22, depressed thereafter. Dorsal integument rugose, weakly areolate, especially along chaetigers 4–16 (Fig. 2A).

Prostomium blunt, three times wider than long. Palps with palpophores massive, directed ventrally, palpostyles digitate, with a basal oblique mark; interpalpal area distinct, right longitudinal depression better defined than left one, expanded posteriorly. Antennae tapered, median antenna twice as long as laterals, laterals barely surpassing palp tips, median antenna reaching chaetiger 2–3. Lateral antennal depressions distinct, more or less parallel to anterior margin of tentacular segment.

Pharynx barely exposed (Fig. 2B). Basal ring not exposed. Distal margin with 14 papillae, four centrolateral ones twice larger than others, each papilla prismatic, tips globular, variably developed.

Tentacular segment three times wider than long; dorsal tentacular cirri slightly longer than ventral ones, approximately half as long as dorsal cirri of chaetiger 1.

Parapodial cirri tapered throughout body. Dorsal cirri tapered, not basally expanded, longer than ventral ones (Fig. 2C, D). Ventral cirri shorter than neurochaetal lobes in anterior chaetigers, as long as neurochaetal lobes in median and posterior chaetigers, missing on chaetiger 2. Prechaetal lobe truncate, not projected along its upper margin, postchaetal lobes long, acute. Anterior and median parapodia with hypertrophied gonopores, some showing abundant sperm (Fig. 2D, inset).

Notochaetae include dorsal hooks from chaetiger 4, heads of hooks fully exposed initially, progressively larger and exposing handles; one accessory capillary present from chaetiger 13. Neurochaetae include three or four short wide pectinates, approximately ten large thin pectinates, and many long finely denticulate capillaries.

Posterior region missing. In paratype tapered to a blunt cone (Fig. 2E). Pygidium laterally expanded, anus terminal, anal cirri ventrolateral, as long as last five or six chaetigers.

Oocytes not seen in parapodial spaces.

###### Etymology.

This species is named after Oscar Díaz-Díaz, Venezuelan polychaete specialist, working now in Chile, as a modest homage to his many publications on polychaetes, and especially because he sampled and processed the specimens including this newly described species. The specific epithet is a noun in the genitive case (ICZN 1999, Art. 31.1.2).

###### Variations.

The paratype was complete. It is 9.5 mm long with 70 chaetigers, but last three chaetigers are hookless. The median antenna is twice as long as the laterals, and the tentacular cirri reach chaetiger 2 or 3 as in the holotype. In the paratype the dorsal hooks start on chaetiger 4, as in the holotype, but although in the holotype the additional capillary is seen from chaetiger 13, in the paratype they are visible from chaetiger 24.

###### Remarks.

*Sigambra diazi* sp. nov. resembles *S. tentaculata* (Treadwell, 1941) and it has been identified as such in previous studies, but the redescription by Moreira and Parapar (2002) of *S. tentaculata* helps for clarifying their differences. They differ in the relative shape of dorsal cirri, and in the size of ventral cirri in comparison to neurochaetal lobe. In *S. diazi* dorsal cirri are tapered, without basal enlargement, and ventral cirri in median and posterior chaetigers are as long as neurochaetal lobes, whereas in *S. tentaculata*, dorsal cirri are basally widened, and ventral cirri are shorter than neurochaetal lobes along body.

###### Distribution.

Only known from the southern Caribbean coast of Venezuela, in shallow muddy bottoms.

##### Sigambra grubii

Taxon classificationAnimaliaAnnelidaPilargidae

Müller, 1858

E6A0B2B0-74C1-5445-91B9-AB66D3B3CECE

Figs 3 , 4

Sigambra grubii Müller, 1858: 214–215, pl. 6, figs 7–9; Pettibone 1966: 182, fig. 13a–c (reinstatement); Salazar-Vallejo 1990: 508–511, figs 1, 2, 4A–C, table 1 (redescription, key to species); Hartwich 1993: 104 (1 syntype).
Sigambra grubei : Licher and Westheide 1997: 3 (new orthography).

###### Material examined.

**Brazil.** Pontal da Daniela (27°27'11"S, 48°31'47"W), in the Santa Catharina Island, Florianópolis. One specimen, 13RC1, July 2018 [mature female, anterior fragment (6.5 mm long, 0.9 mm wide, 54 chaetigers, first dorsal hooks from chaetiger 17)]. One specimen, S1R2, Feb. 2018 [anterior fragment (3.5 mm long, 0.5 mm wide, 27 chaetigers; first dorsal hooks from chaetiger 14)]. One specimen, S2R2C1, Feb. 2018 [anterior fragment (3.7 mm long, 0.8 mm wide, 26 chaetigers; first dorsal hooks from chaetiger 21)]. One specimen, S3R1C1, Feb. 2018 [postlarva (1 mm long, 0.2 mm wide, 13 chaetigers; first dorsal hooks from chaetiger 9)]. Four specimens for SEM (ECOSUR).

###### Description.

**Figure 3.**
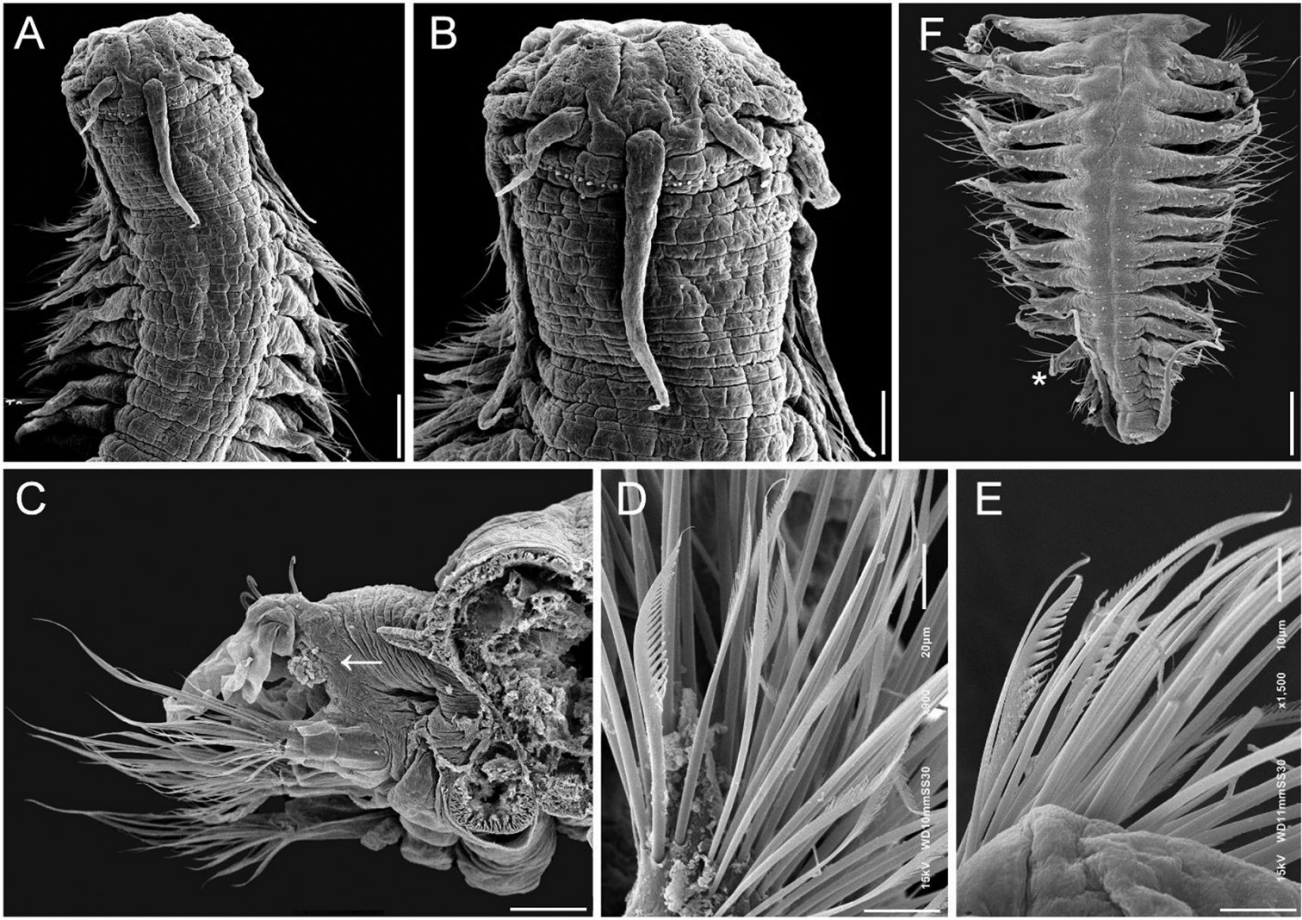
*Sigambra grubii* topotype, small specimen (ECOSUR SEM P1) **A** anterior region, dorsal view **B** same, close-up of anterior end **C** right parapodium, median chaetiger, of larger specimen (6.5 mm long), anterior view (arrow points to gonopore) **D** same, pectinate chaetae **E** same, another parapodium, pectinate chaetae **F** same, posterior region, dorsal view (asterisk indicates last chaetiger with notohooks). Scale bars: 200 µm (**A, F**), 110 µm (**B**), 100 µm (**C**), 22 µm (**D**), 12 µm (**E**).

**Figure 4.**
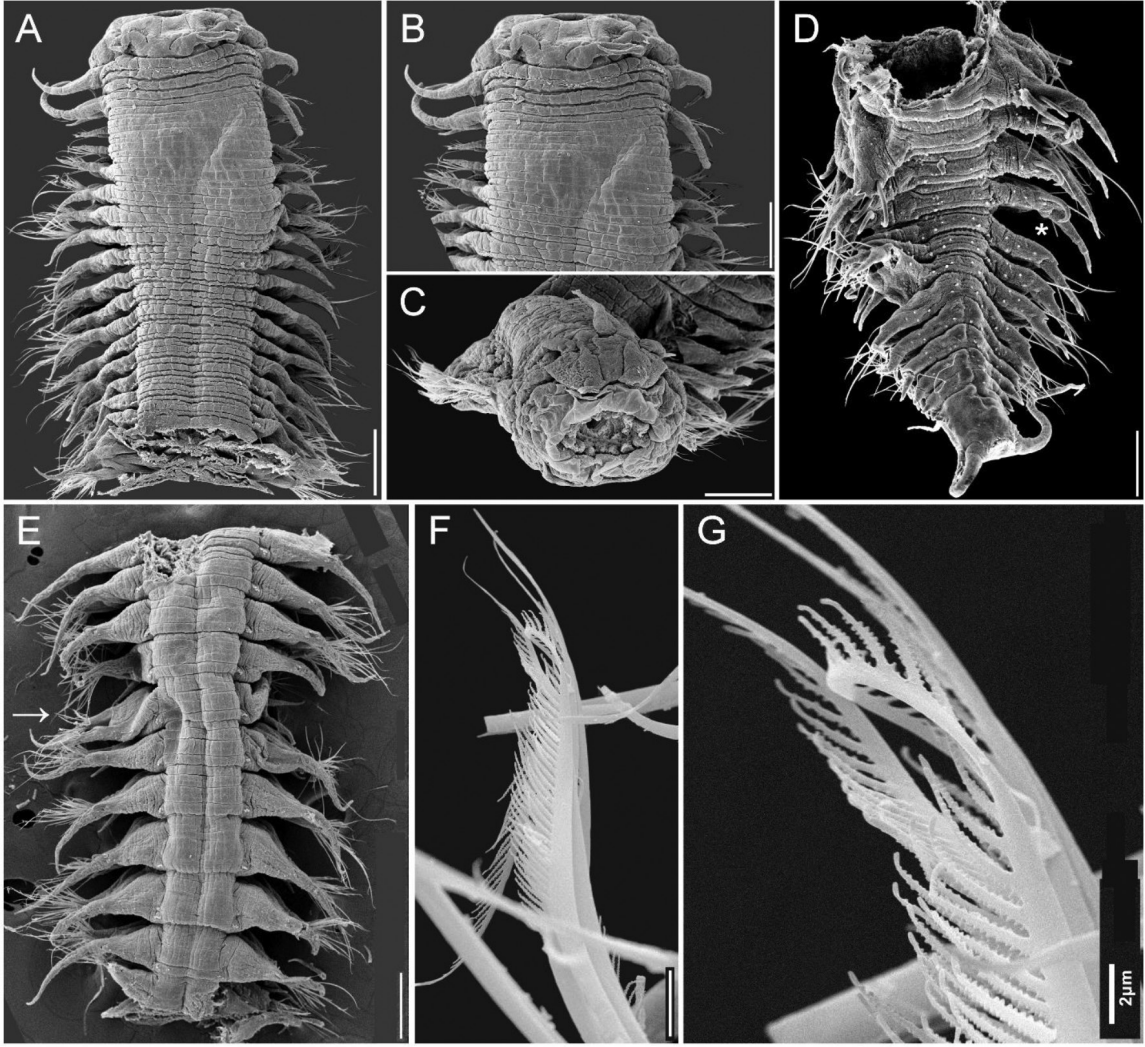
*Sigambra grubii* topotypes, large specimen (ECOSUR SEM P1) **A** anterior region, dorsal view **B** same, close-up of anterior end **C** another specimen, anterior end, frontal view **D** same, posterior region (asterisk indicates last chaetiger with notohooks) **E** specimen in A, median chaetigers (arrow points enlarged chaetae) **F** pectinate chaetae **G** same, detail showing bipinnate denticulation. Scale bars: : 280 µm (**A**), 250 µm (**B**), 170 µm (**C**), 110 µm (**D**), 230 µm (**E**), 8 µm (**F**), 2 µm (**G**).

Prostomium blunt, two or three times wider than long (Figs 3A, B, 4A, B). Palpophores massive, palpostyles barely projected (Fig. 4C); interpalpal area distinct, anteriorly depressed, slightly expanded posteriorly. Antennae tapered, median one two or three times longer than laterals, laterals surpassing tips of palps, median antenna reaching chaetiger 6 or 7. Lateral antennal depressions indistinct.

Pharynx not exposed (Fig. 4C), with 14 thick, prismatic papillae, four lateral ones larger; tips indistinct. Basal pharynx apparently smooth.

Tentacular segment 4–5 times wider than long, with a single transverse row of globular tubercles, with two or three additional short middorsal series; dorsal tentacular cirri slightly longer than ventral ones, as long (Fig. 4A) or half as long (Fig. 3A, B) as dorsal cirri of chaetiger 1.

Parapodial cirri tapered throughout body. Dorsal cirri slightly expanded basally, longer than ventral ones. Ventral cirri as long as neurochaetal lobes in anterior and median chaetigers, longer in posterior ones, missing on chaetiger 2. Prechaetal lobes truncate, projected along its upper margin (Fig. 3C), postchaetal lobes long, acute. Anterior and median parapodia with hypertrophied gonopores, margin granulose (Fig. 3C, arrow).

Notochaetae include dorsal hooks from chaetiger 9–21 (size related), barely exposed initially, handles progressively exposed, without accessory capillaries. Neurochaetae include two or three supracicular shorter wider pectinates (Fig. 4F, G), sometimes with tiny paired denticles along each main tooth, approximately five infra-acicular narrower pectinates (Fig. 3D, E), and abundant finely denticulate capillaries (Fig. 3E).

Posterior region tapered into a truncate cone (Figs 3F, 4D); last 4–7 chaetigers without hooks. Pygidium with two ventrolateral anal cirri, as long as last eight chaetigers.

###### Remarks.

As indicated in the key below, *Sigambra grubii* Müller, 1858 resembles *S. hernandezi* sp. nov. by having dorsal hooks without accessory capillaries. Their main differences are in the size of median antenna, and in the number of posterior chaetigers without hooks. In *S. grubii* the median antenna is medium-sized, reaching chaetiger 3 or 4, and there are 4–6 posterior chaetigers without hooks, whereas in *S. hernandezi* the median antenna is shorter, reaching chaetiger 2 or 3, and there are only two posterior hookless chaetigers. Another subtle difference is the relative size of papillae along body: which are larger in *S. grubii* compared to *S. hernandezi*.

###### Distribution.

The species was described from Florianópolis, southern Brazil, and although it has been reported from a wide bathymetric range along the Brazilian coast, further investigations of this material might show the species to be restricted to shallow water sediments. An interesting fact about Ilha do Desterro (type locality), former name for Florianópolis is the origin of the name. Florianópolis was meant to be a tribute to Marshal Floriano Peixoto, the second President (1891–1894) of the Republic of the United States of Brazil, by combining the first name with the Greek term *polis*, meaning city. Up to 1893, the city was called Nossa Senhora do Desterro (Our Lady of Banishment) or simply Desterro.

##### Sigambra hernandezi sp. nov.

Taxon classificationAnimaliaAnnelidaPilargidae

9BD2E45B-243A-57EA-B515-1027D6D5238F

http://zoobank.org/366BBD1A-5C74-4AAC-B2E2-C01DAF53053C

Figs 5 , 6 , 7

Sigambra grubii : Liñero-Arana and Díaz-Díaz 2005: 68, fig. 1 (**non**Müller 1858).

###### Type material.

***Holotype*** (ECOSUR 216), and ***paratypes* (8)** (ECOSUR 217), Northwestern Caribbean, Chetumal Bay, Quintana Roo, México, Cayo Venado (18°45'04.51"N, 88°06'58.81"W), sandy substrate, 1.5 m, 5 Aug. 2004, J.A. Hoil-Baeza, coll.

###### Additional material.

**Gulf of México.** Tamiahua Lagoon, Veracruz, México, collected by J. A. de León González and M. E. García Garza. One specimen (UANL 4048), collapsed, 4 Nov. 1999 (6.5 mm long, 0.5 mm wide, 86 chaetigers, first dorsal hooks from chaetiger 24). Two specimens for SEM. One mature (UANL 4047), breaking into two parts, Sta. 1-09, 5 Nov. 1999 (11.5 mm long, 0.8 mm wide, 104 chaetigers, first dorsal hooks from chaetiger 14). Another one (UANL 5799), 23 Aug. 2002 (13 mm long, 0.9 mm wide, 103 chaetigers; first dorsal hooks from chaetiger 26). 131 specimens: One, Sta. T-02, 21°42'01"N, 97°35'54"W, 2.2 m. One, Sta. T-09, 21°36'10"N, 97°37'39"W, 2.5 m, 4 Nov. 1999. Three, Sta. T-02, 21°42'01"N, 97°35'54"W, 2.8 m, 7 Mar. 2000; Two, Sta. T-01, 21°42'01"N, 97°39'00"W, 2.8 m; Three, Sta. T-02, 21°42'01"N, 97°35'54"W, 2.8 m; three, Sta. T-04, 21°42'01"N, 97°32'52"W, 2.8 m: One, Sta. T-09, 21°36'10"N, 97°37'39"W, 3 m; one, Sta. T-10, 21°36'10"N, 97°34'45"W; one, Sta. T-11, 21°36'10"N, 97°32'09"W, 4 m; one, Sta. T-12, 4 m, 16 Aug. 2000; One, Sta. T-01, 21°42'01"N, 97°39'00"W, 3 m; three, Sta. T-02, 21°42'01"N, 97°35'54"W, 2.5 m; four, Sta. T-04, 21°42'01"N, 97°32'52"W, 1.5 m; three, Sta. T-05, 21°38'47"N, 97°39'13"W, 2 m; one, Sta. T-07, 21°38'47"N, 97°34'01"W, 3.5 m; two, Sta. T-08, 21°38'47"N, 97°31'14"W, 2.8 m; one, Sta. T-09, 21°36'10"N, 97°37'39"W, 2.5 m; one, Sta. T-10, 21°36'10"N, 97°34'45"W, 3.5 m; five, Sta. T-12, 21°36'10"N, 97°28'59"W, 3.8 m; four, Sta. T-13, 21°32'09"N, 97°36'04"W, 2 m; one, Sta. T-15, 21°32'09"N, 97°30'47"W, 2.5 m; one, Sta. T-16, 21°32'09"N, 97°27'19"W, 2.5 m, 25 Nov. 2000; four, Sta. T-01, 21°42'01"N, 97°39'00"W, 3.5 m; six, Sta. T-02, 21°42'01"N, 97°35'54"W, 2.5 m; one, Sta. T-05, 21°38'47"N, 97°39'13"W, 2.5 m; one, Sta. T-06, 21°38'47"N, 97°36'46"W, 3.8 m; six, Sta. T-07, 21°38'47"N, 97°34'01"W, 4 m; one, Sta. T-08, 21°38'47"N, 97°31'14"W, 2.5 m; five, Sta. T-09, 21°36'10"N, 97°37'39"W, 2 m; two, Sta. T-10, 21°36'10"N, 97°34'45"W, 3 m; five, Sta. T-11, 21°36'10"N, 97°32'09"W, 3.5 m; five, Sta. T-12, 21°36'10"N, 97°28'59"W, 3.5 m; eight, Sta. T-13, 21°32'09"N, 97°36'04"W, 2 m; four, Sta. T-14, 21°32'09"N, 97°32'48"W, 2.5 m; one, Sta. T-15, 21°32'09"N, 97°30'47"W, 2 m; one, Sta. T-16, 21°32'09"N, 97°27'19"W, 3 m, 12 Jul. 2001; three, Sta. T-01, 21°42'01"N, 97°39'00"W, 2.5 m; four, Sta. T-02, 21°42'01"N, 97°35'54"W, 2 m; two, Sta. T-03, 21°42'01"N, 97°34'19"W, 2.5 m; one, Sta. T-04, 21°42'01"N, 97°32'52"W, 1.8 m; two, Sta. T-05, 21°38'47"N, 97°39'13"W, 2 m; two, Sta. T-06, 21°38'47"N, 97°36'46"W, 2.2 m; three, Sta. T-07, 21°38'47"N, 97°34'01"W, 2.6 m; one, Sta. T-09, 21°36'10"N, 97°37'39"W, 2 m; one, Sta. T-10, 21°36'10"N, 97°34'45"W, 2.8 m; five, Sta. T-12, 21°36'10"N, 97°28'59"W, 3 m; one, Sta. T-13, 21°32'09"N, 97°36'04"W, 1 m; two, Sta. T-14, 21°32'09"N, 97°32'48"W, 2.5 m; one, Sta. T-15, 21°32'09"N, 97°30'47"W, 2 m; three, Sta. T-16, 21°32'09"N, 97°27'19"W, 2 m, 23 Feb. 2002. **Chetumal Bay, Q. Roo, México.** Two specimens (ECOSUR), N off Isla Tamalcab (18°38'30.45"N, 88°11'12.84"W), 1 m, sand, 1 Oct. 1996, P. Salazar-Silva, coll. (both complete, 9–10 mm long, 1.1–1.4 mm wide, 69–77 chaetigers; first dorsal hooks from chaetiger 22–28; two last chaetigers hookless). Three specimens (ECOSUR), Río Hondo (18°29'21.99"N, 88°18'32.97"W), sandy mud, 1.5 m, 3 Aug. 2004, J.A. Hoil-Baeza, coll. (anterior fragments, 3.0–3.5 mm long, 0.3–0.5 mm wide, 7–26 chaetigers; dorsal hooks from chaetiger 13–15). Seven specimens (ECOSUR), Punta Amainada (18°42'21.64"N, 88°09'12.36"W), sandy substrate, 1.5 m depth, 4 Aug. 2004, J.A. Hoil-Baeza, coll. (1.7–7.0 mm long, 0.2–0.4 mm wide, 29–68 chaetigers; dorsal hooks from chaetiger 10–32). Twelve specimens (ECOSUR), Cayo Venado (18°45'04.51"N, 88°06'58.81"W), sandy substrate, 1.5 m, 5 Aug. 2004, J.A. Hoil-Baeza, coll. (1.8–11.3 mm long, 0.2–0.8 mm wide, 25–63 chaetigers; dorsal hooks from chaetiger 11–28). Five specimens for SEM (ECOSUR), Cayo Venado, sandy substrate, 1.5 m, 5 Aug. 2004, J.A. Hoil-Baeza, coll. (1.7–6.5 mm long, 0.2–0.4 mm wide, 25–70 chaetigers; dorsal hooks from chaetiger 11–23). **Southwestern Caribbean, Venezuela**. Chacopata or El Maguey lagoons, seven specimens (ECOSUR), 2 m, 15 Feb. 2000, M. Liñero & O Díaz-Díaz, coll. (damaged, some without posterior region, antennae or tentacular cirri broken; smallest specimen with subdermal eyespots in bases of lateral antennae; 4.5–13.0 mm long, 1–2 mm wide, 56–102 chaetigers, first dorsal hooks from chaetiger 13–24).

###### Diagnosis.

*Sigambra* with median antenna reaching chaetiger 2–3, twice as long as laterals; tentacular segment 4 times wider than long, anterior margin smooth; dorsal cirri larger than ventral ones; chaetiger 2 without ventral cirri; notopodia with dorsal hooks from chaetiger 4–5, without capillaries; posterior region with two hookless chaetigers; pharynx with 13–16 marginal papillae.

###### Description.

**Figure 5.**
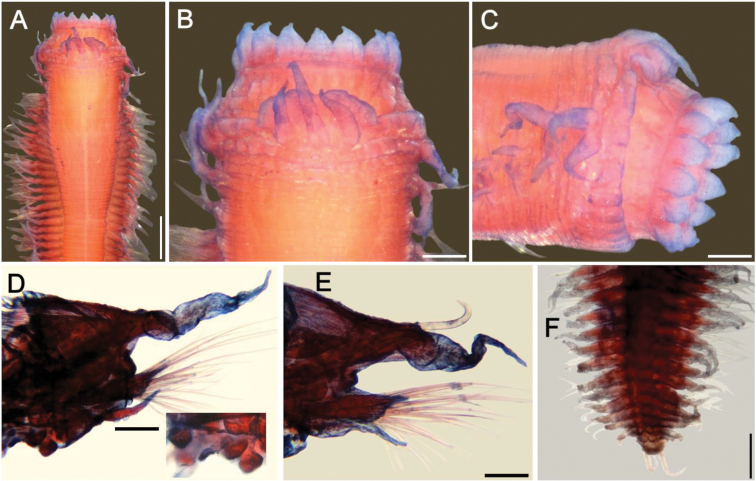
*Sigambra hernandezi* sp. nov., holotype (ECOSUR 216), stained with Shirlastain-A **A** anterior region, dorsal view **B** anterior end, dorsal view **C** same, right lateral view **D** chaetiger 19, right parapodium, posterior view (inset: close-up of oocytes exposed after body wall fracture) **E** chaetiger 62, right parapodium, posterior view **F** posterior region, dorsal view. Scale bars: 0.6 mm (**A**), 0.2 mm (**B, C**), 140 µm (**D**), 80 µm (**E**), 0.3 mm (**F**).

**Figure 6.**
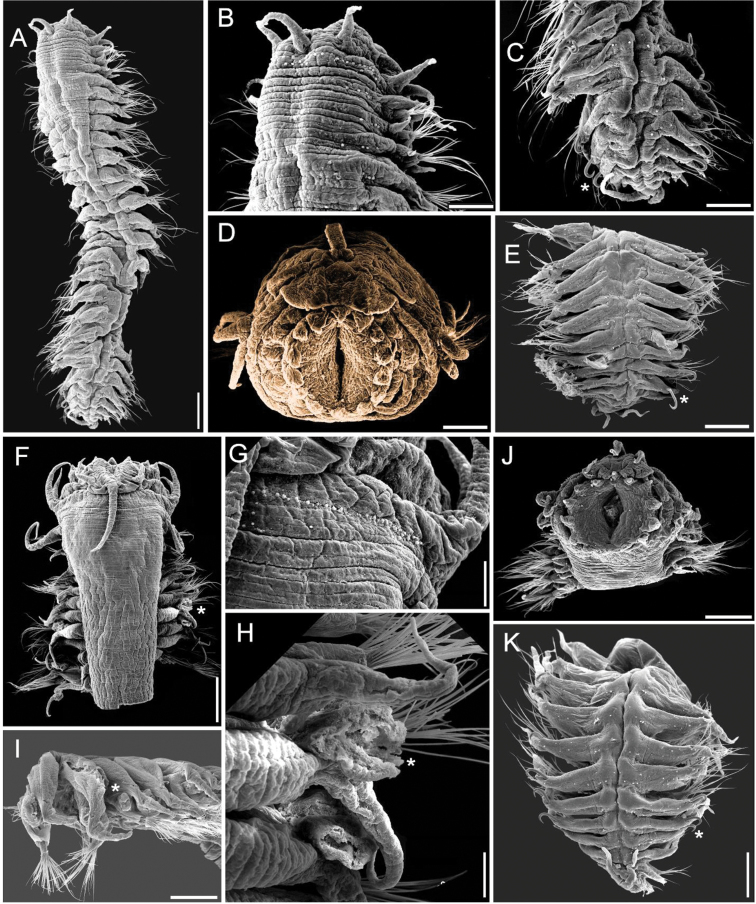
*Sigambra hernandezi* sp. nov. **A** small specimen, dorsal view, body dehydrated **B** same, anterior end, oblique dorsal view **C** same, posterior region, dorsal view (asterisk indicates last chaetiger with hooks) **D** another specimen, anterior end, frontal view **E** another specimen, posterior region, dorsal view (asterisk indicates last chaetiger with hooks). largest specimen (UANL 5799) **F** Anterior region, dorsal view (asterisk indicates globular structures enlarged in **C**) **G** same, close-up of papillae series in tentacular segment **H** same, close-up of interramal globular structures, outer wall broken **I** left parapodia, chaetigers 11–16, oblique frontal view (asterisk indicates gonopore) **J** another specimen (UANL 4047), anterior end, oblique frontal view **K** same, posterior region, dorsal view (asterisk indicates last chaetiger with hooks). Scale bars: 150 µm (**A**), 80 µm (**B, C**), 140 µm (**D**), 170 µm (**E**), 330 µm (**F**), 90 µm (**G**), 60 µm (**H**), 200 µm (**I**), 220 µm (**J**), 140 µm (**K**).

Holotype (ECOSUR 216) slightly twisted along posterior region. Body contracted, cylindrical anteriorly, depressed medially and posteriorly, 9 mm long (paratypes 2.7–9.0 mm long), 2 mm wide (paratypes 0.6–2.0 mm wide), 76 chaetigers (paratypes with 33–82 chaetigers); right parapodia of chaetigers 19, 36, 37, and 62 removed for observing parapodial features. Dorsal integument smooth along chaetigers 1–9 (Figs 5A, 6A), areolate along chaetigers 10–22, smooth, annulated medially and posteriorly.

Prostomium blunt, 2.5 times wider than long (Fig. 5B). Palps with palpophores massive, as long as wide, palpostyles minute, short, barely exposed; interpalpal area indistinct. Antennae tapered, median antenna twice as long as laterals (smaller in smallest specimen, Fig. 6B, larger in largest specimen, Fig. 6F), laterals surpassing palps anterior margin, median antenna reaching chaetiger 2 or 3. Lateral antennal depressions indistinct.

Pharynx fully exposed (Figs 5B, C, 6D, J), with 14 thick, prismatic papillae of similar size, with a short, globular tip. Basal pharynx ring rugose, without distinct papillae.

Tentacular segment six times wider than long, with a single series of transverse globular papillae, barely duplicate or with a few middorsal papillae (Fig. 6G); dorsal tentacular cirri slightly longer than ventral ones, approx. half as long as dorsal cirri of chaetiger 1.

Parapodial cirri tapered throughout body. Dorsal cirri slightly expanded basally, longer than ventral ones. Ventral cirri as long as neurochaetal lobes in anterior and median chaetigers, longer in posterior ones (Fig. 5D, E), missing on chaetiger 2. Prechaetal lobes truncate, not projected along its upper margin, postchaetal lobes long, acute. Anterior and median parapodia with hypertrophied gonopores, margin smooth (Fig. 6F, H, I).

**Figure 7.**
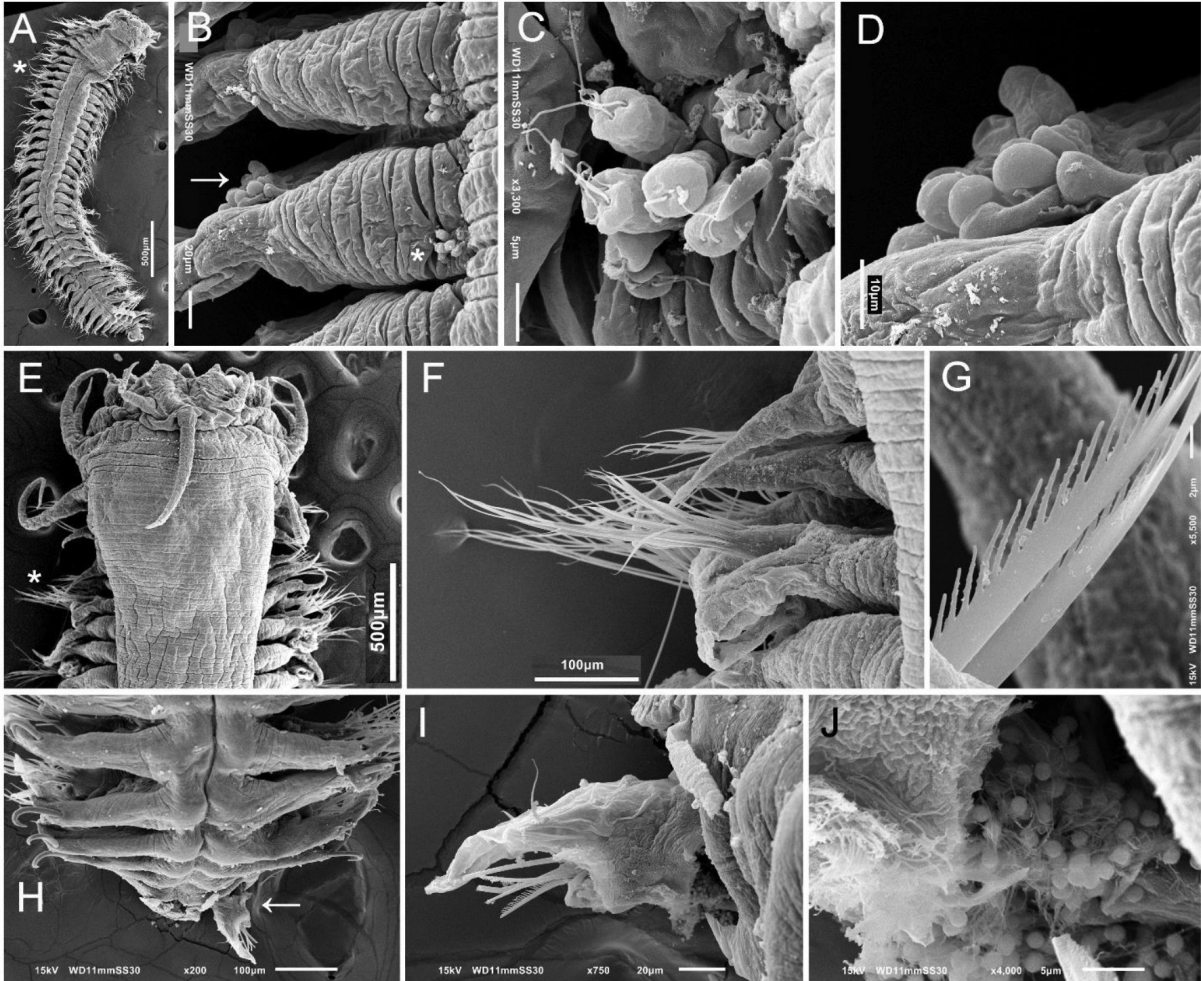
*Sigambra hernandezi* sp. nov. **A** larger specimen, Chetumal, dorsal view (asterisk indicates left chaetigers 8–9) **B** same, chaetigers 8–9, left parapodia, partial dorsal view (asterisk indicates cilia, arrow points to gonopore) **C** chaetiger 9, left parapodium, axillary ciliated papillae, seen from above **D** same, gonopore papillae, seen from above **E** large specimen, Tamiahua, anterior region, dorsal view, asterisk indicates left parapodia of chaetigers 3–4 **F** same, close-up of chaetae, seen from above **G** same, rotated, details of denticles in pectinate chaeta **H** another specimen, posterior region, dorsal view (arrow points to broken posterior chaetiger **I** same, rotated about 90 degrees, showing inner contents **J** same, spermatozoa. Scale bars inserted in micrographs.

Some smaller features are worth mentioning after their observation with SEM. In anterior chaetigers (Fig. 7A) there are some small round papillae or tubercles in the upper, posterior surface of parapodia (Fig. 7B, C), but their tips have 2–5 long cilia, and they probably are sensory structures. The hypertrophied gonopore has a cover of globular smooth, external protuberances (Fig. 7B, D).

Notochaetae include dorsal hooks from chaetiger 28 (in paratypes from chaetigers 11–28), barely exposed initially, handles progressively exposed, without accessory capillaries. Neurochaetae include two or three supracicular, short wide pectinates, 4–6 infra-acicular narrow pectinates, and abundant, long, finely denticulate capillaries.

Posterior region (Figs 5F, 6C, E, K) tapered into a small blunt cone; last two chaetigers hookless. Pygidium with two ventrolateral anal cirri, as long as last 2–4 chaetigers.

Oocytes inside parapodial spaces (Fig. 5D, inset), ca. 100 µm in diameter.

###### Variation.

Specimens were 1.7–13.0 mm long, 0.2–2.0 mm wide, 25–104 chaetigers, with dorsal hooks starting on chaetiger 10–28 with a posterior displacement in larger specimens. The specimens from Tamiahua were slightly larger than those in Chetumal (up to 13 mm long, 104 chaetigers) but because the dorsal hooks start from chaetiger 24–26 whereas they start from chaetiger 14 in the smallest specimen (6.5 mm long), they are regarded as belonging to the same species as the one from Chetumal (up to 11.3 mm long, 76 chaetigers, dorsal hooks from chaetiger 26–28; specimens 6–7 mm long had dorsal hooks on chaetiger 19–26). There were no accessory capillaries with notohooks. The supracicular, wider pectinates (Fig. 7E–G) have small lateral, paired denticles, when compared to those present in *S. grubii*. Oocytes and sperm are present along coelom, but in the posterior region, there are mostly spermatids (Fig. 7H–J), each ca. 1.3 µm in diameter.

###### Etymology.

This species is named after Dr. Héctor A. Hernández-Arana, quantitative benthic ecologist in ECOSUR-Chetumal, in recognition of his many studies on brackish water environments in southeastern México, and especially because he led the research study where Chetumal Bay specimens were collected. The specific epithet is a noun in the genitive case (ICZN 1999, Art. 31.1.2).

###### Remarks.

*Sigambra hernandezi* sp. nov. resembles *S. grubii* Müller, 1858 because they have dorsal hooks without capillaries. They differ especially in the relative size of median antenna, and in the number of posterior chaetigers without hooks. In *S. hernandezi* the median antenna is short, reaching chaetiger 2–3, and there are two hookless chaetigers in the end of body, whereas in *S. grubii* the median antenna is medium sized, reaching chaetiger 3–4, and there are six hookless posterior chaetigers. Another subtle difference is the relative size of papillae along body, which are smaller in *S. hernandezi* and larger in *S. grubii*.

In Tamiahua Lagoon, *S. hernandezi* was an important member of the colonizing benthic polychaetes, being rare after defaunation and progressively becoming one of the more abundant species (Sánchez-Hernández 2009), along a wide range of salinity (5–30‰). In Chetumal Bay, *S. hernandezi* was not among the most abundant species (Hoil-Baeza 2009), and it was common in brackish water seagrasses (*Halodule* sp.). The specimens included in this study were collected along the western shore of Chetumal Bay, which has the lowest salinity values (Carrillo et al. 2009). The specimens from Venezuela are included here with hesitation because they are in suboptimal conditions, but they have a similar pattern in parapodial development and start of the dorsal hooks.

###### Distribution.

Grand Caribbean region, from Tamiahua Lagoon in northern Veracruz, México, to Chetumal Bay, Quintana Roo, in sediments in shallow brackish water; probably reaching the southeastern Caribbean in similar environments.

##### Sigambra ligneroi sp. nov.

Taxon classificationAnimaliaAnnelidaPilargidae

234A2ED3-6803-5E10-8BE8-7C22C2889855

http://zoobank.org/8AE4464D-22F6-45E8-AD25-3337E85BC40F

Fig. 8

Sigambra wassi : Liñero-Arana and Díaz-Díaz 2005: 69–70, fig. 3 (**non**Pettibone 1966).

###### Type material.

***Holotype*** (ECOSUR 218), southern Caribbean, Venezuela. 15 km west off Barcelona (10°06'50"N, 64°51'20"W), dredge, 22 m, 30 May 2000, I. Liñero-Arana & O. Díaz-Díaz, coll.

###### Diagnosis.

*Sigambra* with median antenna twice as long as laterals, reaching chaetiger 1–2; dorsal cirri larger than ventral cirri; chaetiger 2 with ventral cirri; dorsal hooks from chaetiger 26–28, without capillaries; pharynx with 8 marginal papillae.

###### Description.

**Figure 8.**
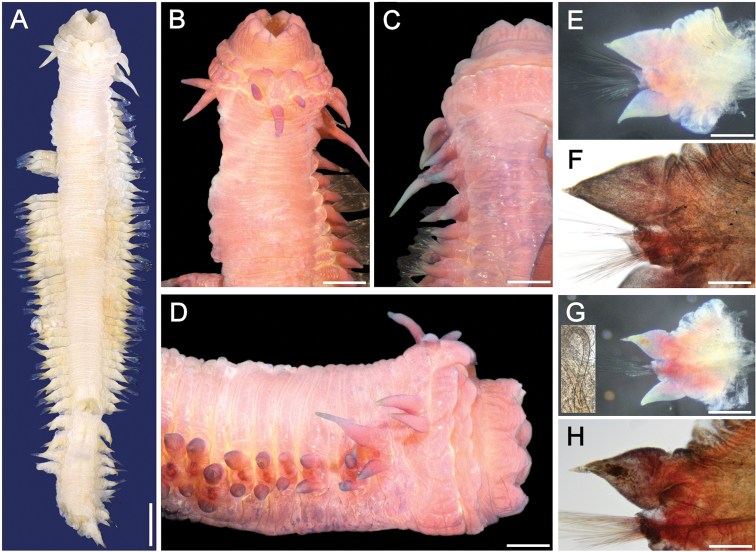
*Sigambra ligneroi* sp. nov., holotype (ECOSUR 218) **A** dorsal view **B** anterior region, dorsal view, after Shirlastain-A staining **C** same, ventral view **D** same, right lateral view **E** chaetiger 16, right parapodium, anterior view **F** same, dorsal cirrus and chaetal lobes **G** chaetiger 36, right parapodium, anterior view (inset: notohook) **H** same, dorsal cirrus and chaetal lobes. Scale bars: 1.4 mm (**A**), 1 mm (**B**), 1.7 mm (**C**), 1.6 mm (**D**), 0.3 mm (**E, G**), 180 µm (**F**), 250 µm (**H**).

Holotype (ECOSUR 218), anterior fragment, bent dorsally, slightly damaged (Fig. 8A). Body contracted, cylindrical anteriorly, depressed medially and posteriorly, 17 mm long, 4 mm wide (excluding chaetae), 49 chaetigers. Dorsal integument rugose, segment margins better defined along first 8 chaetigers, then crenulated along medial and posterior segments. Left parapodia of chaetigers 1–9, 13, 14, and right parapodia of chaetigers 45–49 previously removed. Right parapodia of chaetigers 16 and 36 removed for observing parapodial features.

Prostomium blunt, three times wider than long (Fig. 8B). Palps with palpophores massive, as long as wide, palpostyles tiny, directed laterally; interpalpal area distinct, truncate anteriorly, expanded posteriorly. Antennae digitate, median antenna twice as long as laterals, laterals not reaching tips of palps, median antenna reaching chaetiger 1–2. Lateral antennal depressions indistinct.

Pharynx exposed with two rings (Fig. 8D). Basal ring rugose, with ca. 22 globular projections, better defined dorsally and laterally, basally shorter. Distal ring with 8 short, wide, conical papillae, arranged as 4 per side.

Tentacular segment five or six times wider than long; dorsal tentacular cirri slightly longer than ventral ones, approx. half as long as dorsal cirri of chaetiger 1.

Parapodial cirri tapered along chaetigers 1–4 (5), foliose, longer than wide thereafter; dorsal cirri longer than ventral ones, triangular along anterior chaetigers (Fig. 8E, F), basally wider in posterior ones (Fig. 8G, H), tips long, with globular brownish glands concentrated subdistally, especially along posterior chaetigers. Ventral cirri longer than neurochaetal lobes. Chaetiger 1 with dorsal cirri at least twice as long as ventral ones (Fig. 8C, D). Chaetiger 2 with dorsal cirri slightly longer than ventral ones. Prechaetal lobes rounded, more projected along its upper margin; postchaetal lobes long, acute.

Notochaetae include dorsal hooks from chaetiger 27, barely exposed, without accessory capillaries. Neurochaetae of two types: smaller and medium-sized fine denticulates and long smooth capillaries. No pectinate chaetae present.

Posterior region features unknown. Oocytes not seen.

###### Variation.

A larger specimen was recorded by Liñero-Arana and Díaz-Díaz (2005), and they also provided several illustrations, probably combining some features of both specimens. Their largest specimen was also an anterior fragment but twice as large and with more chaetigers (33 mm long, 5 mm wide, ca. 90 chaetigers). All body features match and they also included an illustration of the far posterior chaetigers; parapodial cirri are tapered, about twice as long as wide, dorsal hooks become more exposed and there is an additional chaeta, thick, slightly falcate, which might correspond to an acicula, instead of a capillary.

###### Etymology.

The species name is after Dr. Ildefonso (Mikel) Liñero-Arana, polychaete specialist from the Universidad de Oriente, Instituto Oceanográfico de Venezuela, Cumaná, in recognition of his many publications about Venezuelan polychaetes. The specific epithet is a noun in the genitive case (ICZN 1999, Art. 31.1.2).

###### Remarks.

*Sigambra ligneroi* sp. nov. resembles *S. wassi* Pettibone, 1966 in having similar body, parapodia and prostomial shapes, such that the holotype plus another specimen were identified as the latter species. They differ, however, in several features, the most important ones being the relative size of antennae, the length of parapodial cirri on chaetiger 1, and the number of marginal pharyngeal papillae, or at least their shape. In *S. ligneroi* the median antenna is twice as long as laterals, dorsal cirri are three times longer than ventral one on chaetiger 1, and there are eight regular papillae on the pharynx. By comparison, in *S. wassi* antennae are subequal, dorsal cirri are twice as long as the ventral ones (cf. Wolf 1984: 29.7, fig. 29.4c), and there are ca. eight irregularly-defined papillae on the pharynx.

As indicated in the key below, *S. ligneroi* also resembles *S. healyae* Gagaev, 2008 because both have ventral cirri on chaetiger 2, and their pharynx has 8 papillae. They differ by the relative size of antennae, and in the start of dorsal hooks; in *S. ligneroi* median antenna is twice as long as laterals, and dorsal hooks start on chaetiger 26–28, whereas in *S. healyae* antennae are subequal and dorsal hooks start on chaetiger 4.

###### Distribution.

Only known from the type locality, off Barcelona, Venezuela, in sediments of water depths of 22 m.

##### Sigambra olivai sp. nov.

Taxon classificationAnimaliaAnnelidaPilargidae

D8464279-03B1-5ACC-8392-833F24421CE9

http://zoobank.org/EF7981AA-9A3A-4BF2-98D1-B324ECE2C478

Figs 9 , 10

###### Type material.

***Holotype*** (ECOSUR 219), Northwestern Caribbean, México, Nichupté Lagoon, NW sector, *Halodule*, Sta. 1 (21°08'55.60"N, 86°47'51.29"W), 1.5 m, 30 Oct 1987, M.S. Jiménez & J.J. Oliva, coll. ***Paratypes* (9)**: One (ECOSUR 220), Nichupté Lagoon, NW sector, *Halodule*, Sta. 1 (21°08'55.60"N, 86°47'51.29"W), 1.5 m, 30 Oct 1987, M.S. Jiménez & J.J. Oliva, coll. One (ECOSUR 221), Nichupté Lagoon, NW sector, *Halodule*, Sta. 1 (21°08'55.60"N, 86°47'51.29"W), 1.5 m, 22 Apr. 1988, M.S. Jiménez & J.J. Oliva, coll. One (ECOSUR 222), Nichupté Lagoon, NE sector, Bojórquez Lagoon, *Halodule*, Sta. 2 (21°07'58.38"N, 86°45'10.39"W), 1.5 m, 27 Oct. 1987, M.S. Jiménez & J.J. Oliva, coll. One (ECOSUR 223), Nichupté Lagoon, NE sector, Bojórquez Lagoon, *Halodule*, Sta. 2 (21°07'58.38"N, 86°45'10.39"W), 1.5 m, 1 Feb. 1988, M.S. Jiménez & J.J. Oliva, coll. Two (ECOSUR 224), Nichupté Lagoon, NE sector, Bojórquez Lagoon, *Halodule*, Sta. 2 (21°07'58.38"N, 86°45'10.39"W), 1.5 m, 5 Jul. 1988, M.S. Jiménez & J.J. Oliva, coll. One (ECOSUR 225), Nichupté Lagoon, NE sector, Bojórquez Lagoon, *Halodule*, Sta. 2 (21°07'58.38"N, 86°45'10.39"W), 1.5 m, 5 Jul. 1988, M.S. Jiménez & J.J. Oliva, coll. Two (ECOSUR 226), Nichupté Lagoon, NE sector, Bojórquez Lagoon, *Thalassia*, Sta. 3 (21°07'01.24"N, 86°45'41.01"W), 1.5 m, 20 Abr. 1988, M.S. Jiménez & J.J. Oliva, coll.

###### Additional material.

**Northwestern Caribbean, México**. One specimen (ECOSUR), Nichupté Lagoon, NE sector, Bojórquez Lagoon, *Thalassia*, Sta. 3 (21°07'01.24"N, 86°45'41.01"W), 1.5 m, 29 Oct. 1987, M.S. Jiménez & J.J. Oliva, coll.

###### Diagnosis.

*Sigambra* with median antenna twice as long as laterals; chaetiger 2 without ventral cirri; dorsal cirri larger than ventral ones; dorsal hooks from chaetiger 30–39, without capillaries; posterior chaetigers without capillary notochaetae; pharynx with 13–16 marginal papillae.

###### Description.

**Figure 9.**
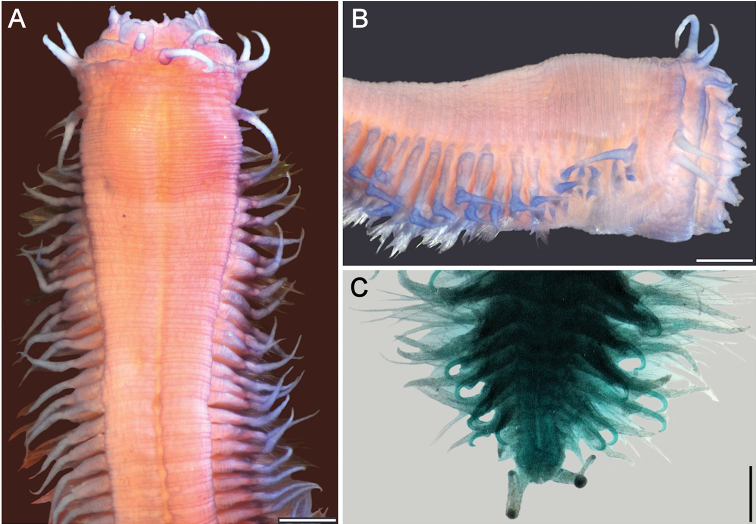
*Sigambra olivai* sp. nov., holotype (ECOSUR 219), stained with Shirlastain-A **A** anterior region, dorsal view **B** anterior region, lateral view **C** posterior end, dorsal view, stained with Methyl green. Scale bars: 1.0 mm (**A**), 0.4 mm (**B**), 0.2 mm (**C**).

**Figure 10.**
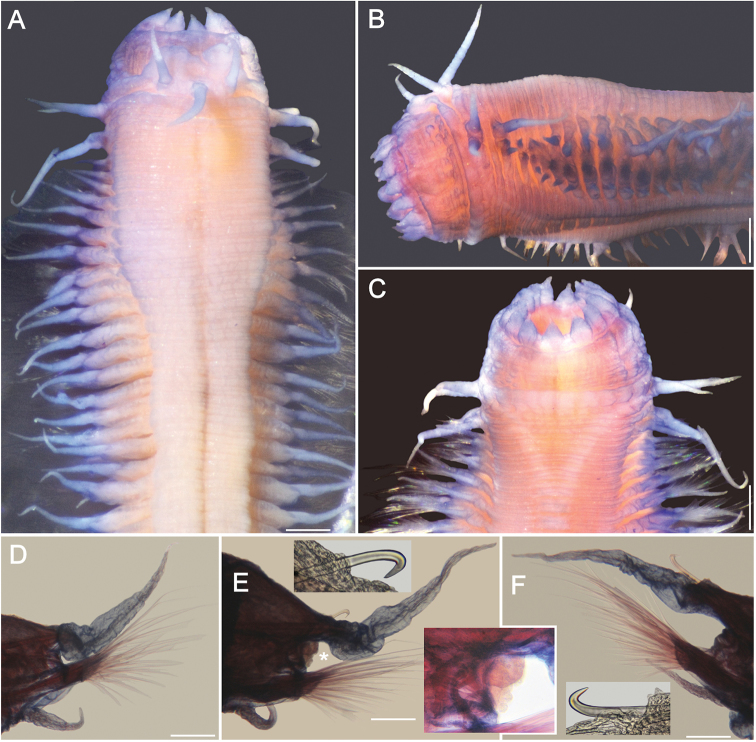
*Sigambra olivai* sp. nov., paratype (ECOSUR 221), stained with Shirlastain-A **A** anterior region, dorsal view **B** same, left, slightly oblique lateral view **C** same, anterior end, ventral view **D** another paratype (ECOSUR 226, complete), chaetiger 30, right parapodium, posterior view **E** same, chaetiger 35, right parapodium, posterior view; asterisk indicates the gonopore (insets: notohook and close-up of interramal gonopore) **F** same, chaetiger 90, right parapodium, anterior view (inset: notohook). Scale bars: 0.3 mm (**A–C**), 0.2 mm (**D**), 150 µm (**E, F**).

Holotype (ECOSUR 219) twisted, broken into two pieces, larger fragment plus posterior end, some median parapodia with hypertrophied gonopores. Body contracted, cylindrical anteriorly, depressed medially and posteriorly, 20 mm long (anterior fragment 14 mm long, posterior one 6 mm long), 2.8 mm wide, 152 chaetigers (90 + 62). Dorsal integument rugose, areolate, especially after chaetigers 6–7 (Figs 9A, 10A). Parapodia removed from paratype.

Prostomium blunt, four times wider than long. Palps with palpophores massive, as long as wide, palpostyles blunt, short, with an oblique basal mark; interpalpal area distinct, blunt anteriorly, expanded posteriorly. Antennae tapered, median antenna twice as long as laterals (left lateral antenna broken), laterals surpassing tips of palps, median antenna reaching chaetiger 2 or 3. Lateral antennal depressions indistinct.

Pharynx with distal ring exposed (Fig. 9A, B), with 15 papillae of similar size, each conical with a globular mucron (rarely duplicated). Basal pharynx ring exposed in one paratype (Fig. 10A–C), with 3–5 series of short, globular papillae, better visible laterally.

Tentacular segment eight or nine times wider than long; dorsal tentacular cirri slightly longer than ventral ones (Figs 9B, 10B, C), about half as long as dorsal cirri of chaetiger 1.

Parapodial cirri tapered throughout body. Dorsal cirri basally expanded, longer than ventral ones. Ventral cirri as long as neurochaetal lobes in anterior and median chaetigers, longer in posterior ones (Fig. 10D–F), missing on chaetiger 2. Prechaetal lobes truncate, slightly projected along its upper margin, postchaetal lobes long, acute. Median parapodia with hypertrophied gonopores, showing distinct globular, papillate or smooth surfaces (Fig. 10E, inset).

Notochaetae only dorsal hooks from chaetiger 38, barely exposed initially, more projected posteriorly, without accessory capillaries. Neurochaetae include two or three supracicular shorter wide pectinates, 4–5 infra-acicular narrow pectinates, especially along anterior and median segments, and abundant, long finely denticulate capillaries.

Posterior region tapered into a small blunt cone; last two chaetigers hookless. Pygidium with two ventrolateral anal cirri (Fig. 9C).

Oocytes inside parapodial spaces, ca. 100 µm in diameter.

###### Variation.

Complete specimens 15–24 mm long, 128–150 chaetigers. Antennae are easily eroded during sieving, but in undamaged specimens the median one is twice as long as lateral ones. Dorsal hooks start in chaetigers 30–39, apparently a size-dependent variation. There are no capillary chaetae with dorsal hooks in posterior chaetigers; last two have no hooks.

###### Etymology.

This species is named after José Juan Oliva-Rivera, amphipod taxonomist in ECOSUR, in recognition of his efforts sampling and processing benthic invertebrates from Nichupté Lagoon, Cancún, México. The specific epithet is a noun in the genitive case (ICZN 1999, Art. 31.1.2).

###### Remarks.

*Sigambra olivai* sp. nov. resembles *S. constricta* (Southern, 1921) by having median antenna twice as long as the laterals, and dorsal hooks from chaetigers 30–40. They especially differ in the presence of a constriction on chaetiger 4, and of capillary notochaetae in posterior chaetigers. In *S. olivai* there is no constriction on chaetiger 4, and there are no capillaries in posterior chaetigers, whereas in *S. constricta* the body has a constriction on chaetiger 4, and there is a single capillary notochaetae in posterior chaetigers.

###### Distribution.

Only known from Nichupté Lagoon, Cancún, México, in seagrasses, mostly *Halodule* sp., in sediments of about 1 m depth.

##### Sigambra wassi

Taxon classificationAnimaliaAnnelidaPilargidae

Pettibone, 1966

8FEB40C1-BA9E-5A20-B30C-40B5B60D7E70

Fig. 11

Sigambra wassi Pettibone, 1966: 186–190, figs 17,18; Wolf 1984: 29-8, fig. 29-4a–j.

###### Type material.

***Holotype*** (USNM 30988), NW Atlantic, Chesapeake Bay, off Rappahanock River (37°37.3'N, 75°59'W), 11 m, sand, Jun. 1962, M. Wass, coll., incomplete posteriorly. ***Paratype*** (USNM 30987), off Rappahanock River (37°37'N, 76°11'W), 13 m, mud, Jul. 1961, M. Wass, coll., broken and in three pieces.

###### Clarification.

**Figure 11.**
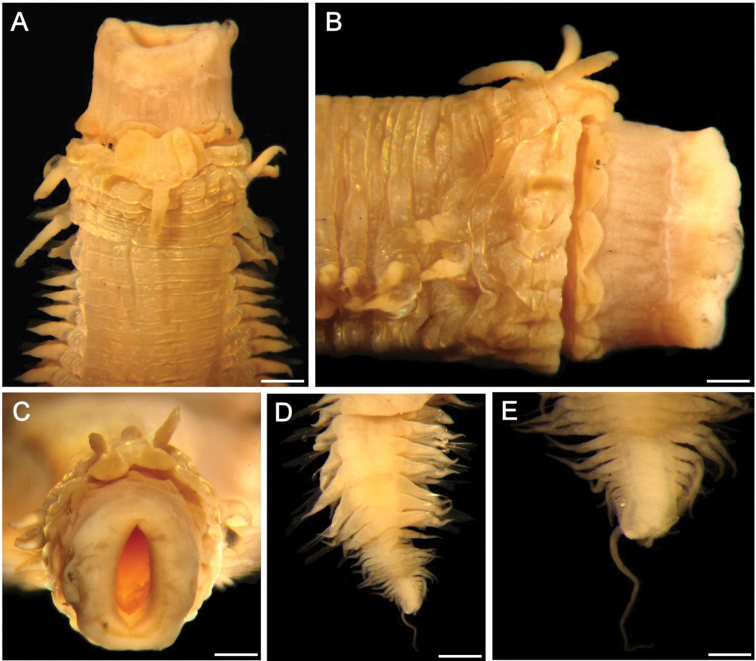
*Sigambra wassi* Pettibone, 1966 **A** holotype (USNM 30988), anterior region, dorsal view **B** same, right lateral view **C** same, frontal view **D** paratype (USNM 30987), posterior region, dorsal view **E** same, posterior end. Scale bars: 0.6 mm (**A, C**), 0.9 mm (**B**), 0.5 mm (**D**), 0.2 mm (**E**).

The original illustrations and descriptions deserve some clarifications. Antennae are certainly of about the same length, but they are not tapered but digitate, and surpass the tips of palps, at least the right one (Fig. 11A). The pharynx is fully exposed, and the basal ring has about 20 irregular hemispherical lobes, but the anterior margin is eroded and damaged (Fig. 11B), such that its marginal papillae are difficult to detect; there are vague indications of the presence of approx. eight large papillae (Fig. 11C). They would include some round projections which might represent the eroded base of papillae, or that they were collapsed when the specimen was compressed into the container.

The posterior end is twisted in the paratype. It had to be pressed by a glass slide in order to have a better observation of the pygidium and anal cirri (Fig. 11D). Anal cirri are delicate, three times longer than pygidial width and tapered (Fig. 11E), not subcylindrical as originally illustrated.

###### Remarks.

After the study of type material, the number of marginal papillae is not defined for *S. wassi* Pettibone, 1966. Their number might be the same as in *S. ligneroi* sp. nov. described above, but better specimens are needed to clarify this. On the other hand, most diagnostic features for the species were confirmed by Wolf (1984), the fragility of anal cirri might explain why they were not observed by him.

#### Key to species of *Sigambra* Müller, 1858^*^

(modified after Salazar-Vallejo 1990 and Licher and Westheide 1997)

**Table:** 

1	Dorsal cirri larger than ventral cirri	**2**
–	Dorsal and ventral cirri subequal	**20**
2	Chaetiger 2 without ventral cirri	**3**
–	Chaetiger 2 with ventral cirri	**21**
3	Pharynx with 8 marginal papillae	**4**
–	Pharynx with 13–16 marginal papillae	**7**
4	Dorsal hooks from chaetigers 3–8	**5**
–	Dorsal hooks from chaetigers 15–17 (5–18 mm long; up to chaetiger 30?); median and posterior notopodia with 1 capillary; median antenna long, reaching chaetiger 7	***S. vargasi* Dean, 1999 (Pacific Costa Rica)**
5	Median and posterior notopodia with capillaries	**6**
–	Notopodia without capillaries; all neurochaetae with tips entire; median antennae reaching chaetiger 5 (17–23 mm long)	***S. papagayu* Bamber in Muir and Bamber 2008 (Hong Kong)**
6	Some neurochaetae with bifid tips; median antenna barely longer than laterals, reaching chaetiger 2; median and posterior notopodia with 2 capillaries	***S. bidentata* Britayev & Saphronova, 1981 (Sea of Japan)**
–	All neurochaetae with tips entire; median antenna markedly longer than laterals, reaching chaetiger 3; median and posterior notopodia with 1 capillary	***S. qingdaoensis* Licher & Westheide, 1997 (Yellow Sea)**
7	Dorsal hooks from anterior chaetigers (4–18)	**8**
–	Dorsal hooks from median chaetigers (30–40); median antenna twice as long as lateral ones, or longer	**19**
–	Dorsal hooks from posterior chaetigers (42–66), or beyond that (14 mm long); median antenna as long as lateral ones, barely reaching chaetiger 1	***S. rugosa* Fauchald, 1972 (Western México)**
8	Tentacular segment about twice wider than long	**9**
–	Tentacular segment 4 times wider than long	**10**
9	Tentacular segment with anterior margin with rounded projected lobes, external to lateral antennae	***Sigambra* sp. indet.** (**Brazil (AER))**
–	Tentacular segment with anterior margin smooth, without projected lateral lobes; dorsal hooks from chaetiger 3–4 (14 mm long)	***S. setosa* Fauchald, 1972 (Western México)**
10	Tentacular segment with rounded projected lobes in anterior margin; median antenna slightly longer than lateral ones, reaching chaetiger 3–4; dorsal hooks from chaetiger 4–5 (6–12 mm long)	***S. parva* (Day, 1963) (South Africa)** ^**^
–	Tentacular segment with anterior margin smooth, without rounded projected lobes	**11**
11	Median and posterior notopodia with capillaries	**12**
–	Notopodia without capillaries	**18**
12	Median antenna short, reaching up to chaetigers 3–4	**13**
–	Median antenna medium-sized, reaching chaetigers 5–7	**14**
–	Median antenna long, reaching chaetigers 9–12; dorsal hooks from chaetigers 11–15 (40 mm long); lateral antennae without lateral depressions	***S. bassi* sensu Blake, 1994 (California)**
13	Dorsal hooks from chaetiger 4 (15 mm long); median antenna slightly longer than laterals; first dorsal cirri slightly longer than dorsal tentacular ones	***S. tentaculata* sensu Blake, 1994 (NE Pacific)**
–	Dorsal hooks from chaetiger 12–18 (16 mm long); median antenna twice as long as laterals; first dorsal cirri markedly longer than dorsal tentacular ones	***S. elegans* Britayev & Saphronova, 1981 (Sea of Japan)**
14	Median antenna slightly longer than lateral ones, reaching chaetiger 4–6; dorsal hooks from chaetiger 7–10 (5.5 mm long)	***S. pettiboneae* Hartmann-Schröder, 1979 (NW Australia)**
–	Median antenna twice as long as lateral ones	**15**
15	Median antenna reaching chaetiger 7–8	**16**
–	Median antenna reaching up to chaetiger 5–6; dorsal hooks from chaetiger 4–5	**17**
16	Dorsal hooks from chaetiger 3–9 (5–20 mm long)	***S. hanaokai* (Kitamori, 1960) (Seto Island, Japan), Reinst. Nishi et al. 2007**
–	Dorsal hooks from chaetiger 12–18 (24 mm long)	***S. bassi* (Hartman, 1947) (Florida)** ^***^
17	All parapodia with ventral cirri shorter than neuropodial lobes; dorsal cirri basally wider	***S. tentaculata* (Treadwell, 1941) (NW Atlantic), Moreira and Parapar 2002**
–	Median and posterior parapodia with ventral cirri long, reaching tip of neuropodial lobes; dorsal cirri tapered, not wider basally	***S. diazi* sp. nov. (southern Caribbean, Venezuela)** ^****^
18	Median antenna medium-sized, reaching chaetigers 3–4; posterior region with 4–6 hookless chaetigers; body papillae large	***S. grubii* Müller, 1858 (southern Brazil)**
–	Median antenna short, reaching chaetiger 2–3; posterior region with 2 hookless chaetigers; body papillae small	***S. hernandezi* sp. nov.** (**NW Atlantic, brackish water)**
19	Median antenna slightly longer than laterals; body without a constriction on chaetiger 4; dorsal hooks from chaetiger 30–39 (15–24 mm long); posterior chaetigers without capillary notochaetae	***S. olivai* sp. nov.** (**Northwestern Caribbean, México)**
–	Median antenna twice as long as laterals; body with a constriction on chaetiger 4; dorsal hooks from chaetiger 30–40 (16–24 mm long); posterior chaetigers with a single capillary notochaetae	***S. constricta* (Southern, 1921) (Northeastern India, brackish water)**
20	Dorsal hooks from chaetiger 6 (1.5 mm long)	***S. ocellata* (Hartmann-Schröder, 1959) (El Salvador, brackish water)**
–	Dorsal hooks from chaetiger 3 (3.7 mm long)	***S. magnuncus* Paterson & Glover, 2000 (NE Atlantic, abyssal)**
21	Pharynx with 8 marginal papillae	**22**
–	Pharynx with 14–16 marginal papillae	**24**
22	Median antenna slightly longer than laterals	**23**
–	Median antenna twice as long as laterals; dorsal hooks from chaetiger 26–28	***S. ligneroi* sp. nov. (southern Caribbean, Venezuela)**
23	Dorsal hooks from chaetiger 4 (0.7 mm wide), tentacular segment as long as wide	***S. healyae* Gagaev, 2008 (Arctic Ocean)**
–	Dorsal hooks from chaetigers 23–30 (45–70 mm long); tentacular segment 4–5 times wider than long	***S. wassi* Pettibone, 1966 (Northwestern Atlantic)**
24	Dorsal hooks from chaetiger 7, with accessory capillaries; dorsal cirri progressively longer from chaetiger 2	***Sigambra* sp. Imajima, 2001 (Eastern Japan)**
–	Dorsal hooks from chaetigers 43–70, without capillary chaetae; dorsal cirri of chaetiger 2 smaller than those in following chaetigers	***S. robusta* (Ehlers, 1908) (Southwestern Africa)**

## Supplementary Material

XML Treatment for Sigambra

XML Treatment for Sigambra bassi

XML Treatment for Sigambra diazi

XML Treatment for Sigambra grubii

XML Treatment for Sigambra hernandezi

XML Treatment for Sigambra ligneroi

XML Treatment for Sigambra olivai

XML Treatment for Sigambra wassi

## Footnotes

The key includes two undescribed species being recorded with other names, or not completely identified, but in both cases, they are distinct and deserve to be described.

Redescr. Moreira and Parapar 2002 (including S. cf. tentaculataKatzmann et al., 1974fideMoreira and Parapar 2002)

N.C. specimens, after Gardiner 1976: 122, fig. 9d, have shorter median antenna

cf. S. bassi sensu Licher and Westheide 1997 Washington
